# Functional Role of Suprahyoid Muscles in Bolus Formation During Mastication

**DOI:** 10.3389/fphys.2022.881891

**Published:** 2022-06-08

**Authors:** Anna Sasa, Sirima Kulvanich, Naohito Hao, Reiko Ita, Masahiro Watanabe, Taku Suzuki, Jin Magara, Takanori Tsujimura, Makoto Inoue

**Affiliations:** ^1^ Division of Dysphagia Rehabilitation, Niigata University Graduate School of Medical and Dental Sciences, Niigata, Japan; ^2^ Department of Community Dentistry and Gerodontology, Faculty of Dentistry, Thammasat University, Klongluang, Thailand; ^3^ Department of Special Needs Dentistry, Division of Hygiene and Oral Health, Showa University School of Dentistry, Tokyo, Japan

**Keywords:** bolus formation, electromyography, suprahyoid muscles, mastication, mandibular kinematics

## Abstract

It still remains unclear how the suprahyoid muscles function in bolus formation during mastication. This study aimed to investigate the contributory role of the suprahyoid muscles during mastication. A total of 20 healthy young volunteers were asked to perform tongue pressure generation tasks and unilateral mastication tasks using peanuts and two different types of rice crackers. Surface electromyographic (EMG) activity of the masseter and suprahyoid muscles and mandibular kinematics were recorded. Suprahyoid activity increased with increasing tongue pressure. Masticatory duration until the first deglutition differed significantly among the different foods; the harder the food, the longer the duration. This was also the case in masseter activity per masticatory cycle. Masticatory rate and suprahyoid activity per masticatory cycle were significantly higher during soft rice cracker mastication. Masseter activity was higher on the masticatory side than on the non-masticatory side, however, there was no difference in suprahyoid activity between the sides. Suprahyoid activity and jaw gape showed significant positive correlation in the early stage on both the masticatory and non-masticatory sides. The suprahyoid muscles functioned dominantly for jaw-opening during peanut mastication, and for bolus formation, especially in the late stage during soft rice cracker mastication. Bolus formation was performed dominantly on the masticatory side during rice cracker mastication. These findings clearly demonstrate a functional role of the suprahyoid muscles during mastication of solid foods from assessments using both EMG activity and mandibular kinematic recordings.

## 1 Introduction

Mastication is essential for the adequate intake of solid foods in most mammals (Hiiemae et al., 1996). During mastication, food is crushed and mixed with saliva by the actions of the teeth and masticatory muscles while the tongue, palate, and cheeks contribute to forming a food bolus. Although underlying digestive motor actions such as mastication and deglutition are triggered and controlled by a central pattern generator in the brainstem (Jean, 2001; Lund and Kolta, 2006), sensory information from the bolus in terms of its size, temperature, texture, or moisture, changes from moment to moment, and motor patterns can adapt to these changing characteristics. Previous studies demonstrated a relationship between masticatory muscle activity and the mechanical properties of food during mastication; masticatory movements are affected by bolus property, primarily the hardness of the food and bolus hardness changed masticatory force, mandibular movement and masticatory cycles, and masticatory cycle times (Horio and Kawamura, 1989; Bishop et al., 1990; Hiiemae et al., 1996; Lassauzay et al., 2000; Peyron et al., 2002; Grigoriadis et al., 2014; Takei et al., 2020).

Once the food is broken down by the masticatory muscles, the differences in these properties decrease, and the changes in food consistency affect the movements required to make the bolus suitable for deglutition. In this regard, some properties of the bolus other than hardness should also be considered. In a previous study, we compared masticatory behaviors between different rice products using steamed rice and rice cake (Iguchi et al., 2015). We found that masseter and suprahyoid electromyographic (EMG) activity per masticatory cycle was higher for rice cake than for steamed rice although the hardness of the boluses was similar throughout the masticatory process. Because cohesiveness and adhesiveness were significantly higher for the latter than the former, we suggested that a difference in cohesiveness also has a critical effect on masticatory performance as previously reported (Kohyama et al., 2005). In addition, we evaluated masticatory activity using rice crackers with different physical properties (Takei et al., 2020). As expected, the harder/larger the rice cracker, the longer the masticatory duration and the higher the number of masticatory cycles. Conversely, the suprahyoid EMG activity was much higher for the soft rice cracker than for the others. We also found that the water absorption rate of the bolus was significantly higher for the rice cracker with the lowest hardness and density compared with other rice crackers. It would likely have been more difficult to transport the bolus that had high water absorption compared with others in the late stage of the masticatory cycle. In those studies, however, only the average suprahyoid muscle activity was compared among the foods, thus, it remains unclear how the bolus properties affected masticatory behaviors.

As with recordings of EMG activity, numerous studies have assessed the functional contribution of intraoral structures such as the tongue to bolus formation and transport during mastication using videofluorography (Palmer et al., 1992; Palmer et al., 1997). This is because movements of the bolus and intraoral structures cannot be directly visualized. Palmer et al. (1992) reported on bolus propulsion during mastication where the bolus was moved toward the pharynx during the late stage of mastication, that is, stage II transport. The authors also demonstrated that the patterns of mandibular and tongue movements during stage II transport were characterized by exaggerated upward movements of the tongue that compress food against the palate during the jaw-closing phase. The tongue is attached to the hyoid bone and several mastication-related muscles are attached to the hyoid itself including the suprahyoid muscles, which keep the tongue in place. The suprahyoid muscles are also known to be jaw-opening muscles. Therefore, it is plausible that changes in tongue muscle activity may be accompanied by changes in suprahyoid muscle activity, and hence mandibular movements can also change depending on the masticatory stage. Nonetheless, our knowledge of the functional contribution of the suprahyoid muscles to bolus formation and the differences in these activities and mandibular kinematics among foods is rather limited.

This study was designed: 1) to investigate the contribution of the suprahyoid muscles to bolus formation during mastication; 2) to clarify how suprahyoid muscle activity for bolus formation differs among foods; and 3) to elucidate the difference in suprahyoid muscle activity between the masticatory and non-masticatory sides. We hypothesized that the ratio of suprahyoid muscle activity to vertical distance of jaw opening represents the functional role of these muscles in bolus formation, and thus differs depending on bolus properties and masticatory stage.

## 2 Materials and Methods

### 2.1 Participants

This study involved 20 healthy volunteers (12 men, eight women), ranging from 23 to 44 years (average age ±standard deviation [SD], 31.0 ± 6.1 years). Prior to obtaining recordings, an attending dentist confirmed that all participants had no missing teeth except the third molar teeth, no temporomandibular disorder and no masticatory or deglutition problems at meal. Written informed consent was obtained from all participants, and the study was approved by the Ethics Committee of Niigata University (approval no. 2020–0039). All experiments were performed in accordance with the Declaration of Helsinki guidelines for studies involving human participants (2008).

### 2.2 Test Foods

**FIGURE 1:**
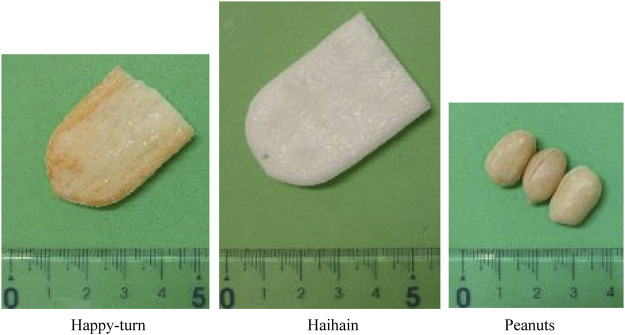
Photograph showing the food samples, Happy-turn (Happy), Haihain, and peanuts (Nuts).

In this study, we used two commercially available rice cracker products and peanuts (Nuts) as test foods (Figure 1). The rice crackers were Happy-Turn (Happy) and Haihain (Kameda Seika Co. Ltd., Niigata, Japan). Peanuts are one of the most common solid foods used in dental research to evaluate masticatory function (Gonçalves et al., 2014; Goto et al., 2015; Yamasaki et al., 2016; Oki et al., 2021). We believe that rice crackers are also a suitable sample food to evaluate masticatory function because they are typically hard and brittle. Eating rice crackers requires many steps of the process in the oral cavity, including function not only of the masticatory muscles but also the tongue and cheek muscles to crush and mix the bolus with saliva. As previously described, the thickness of one piece of Happy and Haihain was 8.9 ± 0.2 mm and 6.4 ± 0.2 mm, and the density was 0.397 ± 0.021 g/ml and 0.115 ± 0.002 g/ml, respectively (Takei et al., 2020). Both properties were significantly higher for Happy than Haihain. The hardness of the food was measured using a creep meter (RE2-33005S, YAMADEN CO.,LTD., Tokyo, Japan). The maximum load was 39.9 ± 13.0 N for Nuts, 26.5 ± 10.5 N for Happy, and 8.7 ± 2.6 N for Haihain. Happy characteristically had a higher fat content (28.9%) than Haihain (1.2%). Mouthful volume for each trial was determined as 3 g except for Haihain, which was 0.85 g. Haihain had a very low density, and so the volume was adjusted to the same volume as that of Happy.

### 2.3 EMG Activity and Videoendoscopic Recordings

The methodology was precisely as described in our previous study (Takei et al., 2020). Briefly, surface EMG activities were recorded from the masseter and suprahyoid muscles on both the left and right sides. Electrodes (NT-611T; Nihon Kohden, Tokyo, Japan) were attached to the skin over the masseter muscle and the anterior belly of the digastric muscle for the suprahyoid muscles. The suprahyoid musculature comprises the geniohyoid, mylohyoid, and the anterior belly of the digastric muscle. Using surface electrode, EMG activities of all these muscles were recorded (Palmer et al., 1999). Signals were filtered and amplified to remove movement-related artifacts (low-pass and high-pass cut-off frequency, 30 Hz and 2 kHz, respectively) (AB-611J; Nihon Kohden).

VE images were recorded to identify deglutition. A fibre-optic endoscope (FNL-10RP3; Pentax, Tokyo, Japan) was inserted through the nasal passage and into the midpharynx. All signals, including EMG and VE data, were stored through an interface board (PowerLab; ADInstruments, Colorado Springs, CO) on a personal computer (2 kHz for EMG and 33 Hz for VE images). Data analysis was performed using the PowerLab software package (LabChart 8; ADInstruments).

### 2.4 Mandibular Kinematics

Mandibular kinematics were recorded using a 10-camera Vicon motion capture system (Vicon Motion Systems, Oxford, United Kingdom), which documented three-dimensional coordinates of reflective markers on the skin covering maxillary and mandibular bones. First, laboratory-fabricated adhesive frame housing markers were placed on the scalp. Next, three reflective markers were placed at specific cephalometric landmarks, namely the nasion and the left and right gonions, in a plane parallel to the Frankfort horizontal plane. Another marker was placed on the pogonion. These signals were stored on a personal computer at 100 Hz and were extracted in csv format using a software program (MATLAB, R2021a; Mathworks, Natick, MA). Finally, mandibular kinematics data was synchronized off-line with EMG activities and VE images using LabChart 8 (ADInstruments).

### 2.5 Data Processing

**FIGURE 2:**
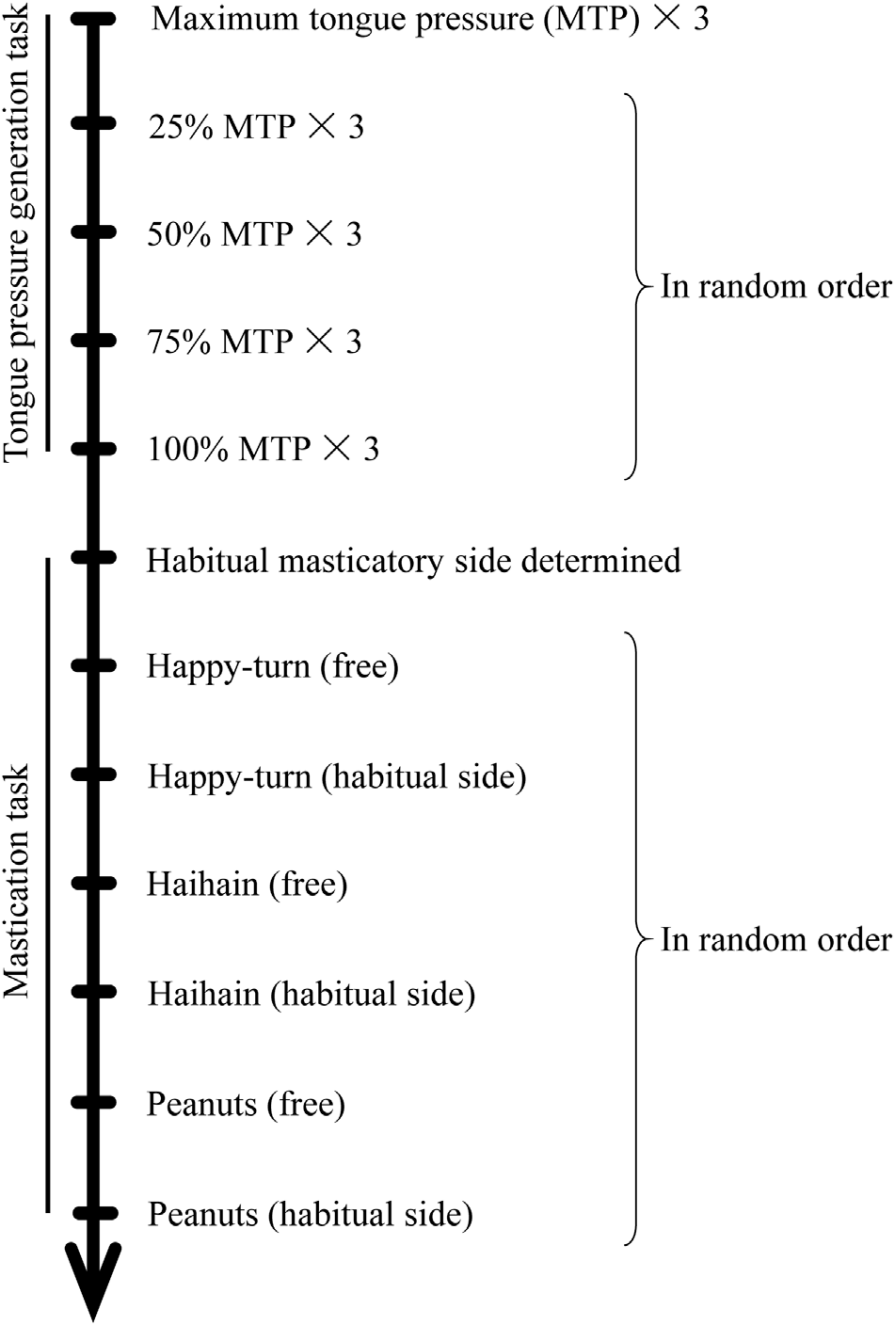
Experimental protocol.

Participants were instructed not to eat or drink for at least 1 h prior to the experiment to avoid the situation in that the participants eat any foods immediately before the experiment and are on a full stomach. They were seated in an upright sitting position without headrests throughout the duration of the experiment. In this study, we conducted two tests involving specific tasks: tongue pressure generation followed by food mastication tasks (Figure 2).

In the tongue pressure generation task, each participant was first asked to maximally open the jaw three times for 5 s each to normalize the suprahyoid EMG activity. They were then asked to press the tongue as hard as possible against the anterior aspect of the hard palate for 7 s each three times; this maximum tongue pressure (100%) was measured by using a balloon-type tongue pressure instrument (JM-TPM02, JMS Co., Japan). To obtain the stable EMG burst in one trial, it took several seconds so that we determined 5-s jaw opening task and 7-s tongue pressure generation. Further, from the three data, we confirmed the EMG activity was reproducible (data not shown).

In the procedure, they lightly held the probe in 4-mm diameter. We confirmed that no apparent EMG activity was observed during only holding the probe. The average of 100% tongue pressure was calculated and then participants were asked to repeat the task at 25, 50, 75, and 100% pressure for 7 s in random order. Visual feedback was provided during recordings and suprahyoid EMG activities on the left and right sides were recorded. An interval of at least 1 min was allowed between trials.

In the masticatory task, participants were asked to unilaterally masticate and then ingest three different types of test foods (Happy, Haihain, and Nuts) in a random order. Subjects were asked to masticate on their preferred/habitual masticatory side that was determine in a previous study (Sano and Shiga, 2021; Shiga et al., 2021), where subjects were asked to masticate on a gummy jelly test food and report the side on which mastication appeared to be easier.

**FIGURE 3:**
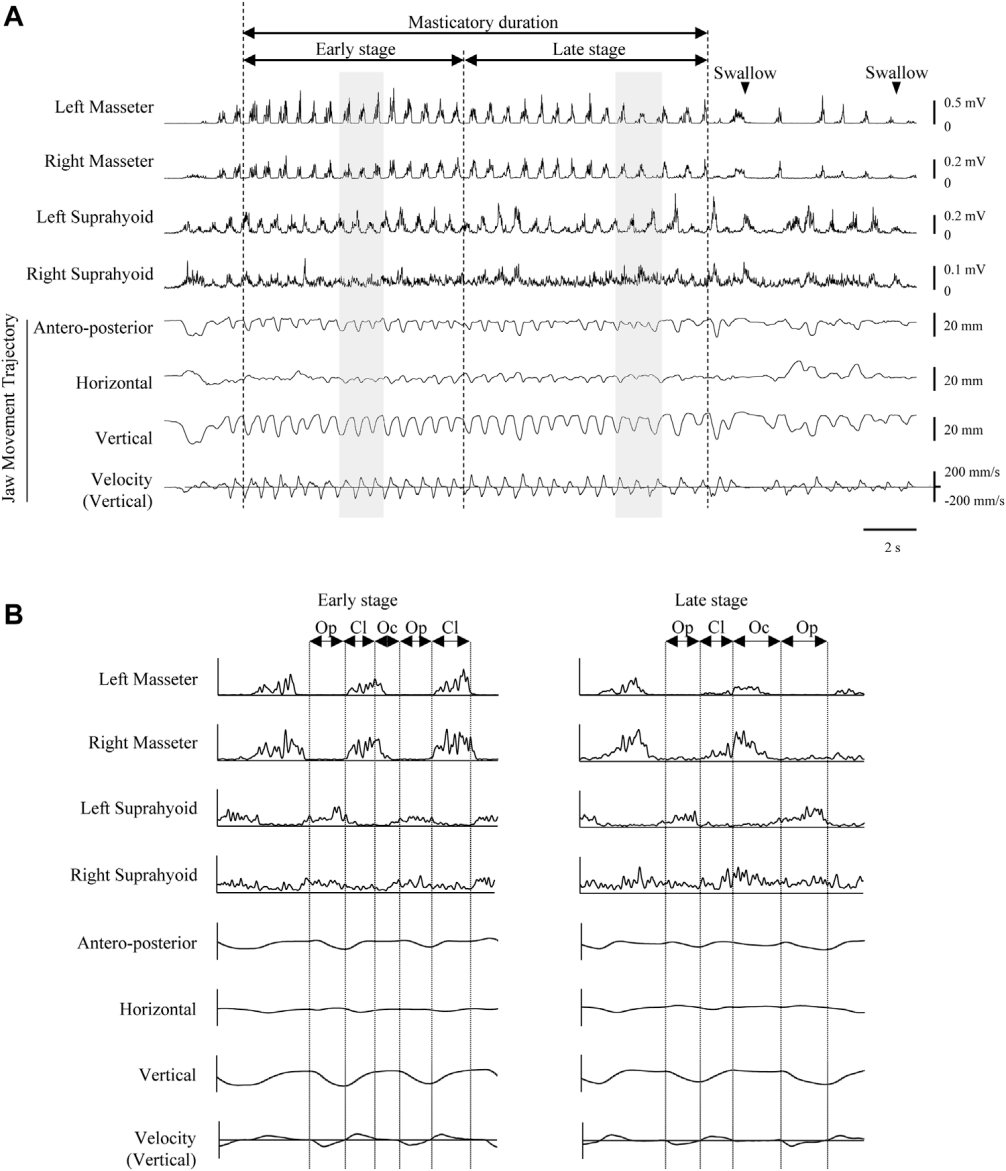
Representative electromyographic (EMG) activity recording and mandibular movement trajectories during Happy mastication on the habitual (right) side. Rectified and smoothed EMG waveforms are shown. **(A)** Vertical dotted lines indicate masticatory onset, border between early and late stages, and offset of masticatory duration. **(B)** Expanded view of shaded areas in recordings A. Reciprocal EMG bursts commonly observed in masseter and suprahyoid muscles on both sides in the early stage. Vertical dotted lines indicate the border between phases. Cl, jaw-closing phase; Oc, occlusal phase; Op, jaw-opening phase.

During these tasks, masseter and suprahyoid EMG activities, VE images, and mandibular kinematics were recorded simultaneously (Figure 3A). Each trial ended with a right hand raise when the participant had finished eating. The interval between trials was set to at least 1 min, and participants could rinse their mouths with water whenever they wished between the trials.

### 2.6 Data Analysis

To determine the threshold of EMG activity, all EMG waveforms were first full-wave rectified and smoothed (time constant 20 ms).

### 2.6.1 Tongue Pressure Generation Task

Using the data recorded during both maximum jaw-opening and tongue pressure generation, the mean amplitude of the area under the curve of the rectified suprahyoid EMG activities for 1 s, which was obtained by averaging the left and right EMG data, was calculated for each task (25, 50, 75, and 100%). Regarding the relationship between the maximal mouth opening and the position of the mandibular condyle, when the maximal mouth opening is wide, the mandibular head shifts towards the anterior, and next the condyle is located anterior to the articular tubercle. This suggests that not only on the jaw distance but also the size of the mandibula affects the mandibular head/condyle movement and suprahyoid EMG activity. Because mouth-opening capacity was different among the participants, we decided to use the EMG values normalized to those recorded maximum jaw-opening. Normalized mean activity of suprahyoid EMG burst was compared using one-way repeated measures ANOVA followed by Tukey’s honestly significant difference (HSD) test for further analysis.

### 2.6.2 Mastication Task

During mastication, each masticatory cycle had three components, namely the jaw-opening, jaw-closing, and occlusal phases, which were determined based on the speed and direction of the vertical and horizontal jaw movements, respectively (Figure 3B). The jaw-opening phase began at the uppermost mandibular position and ended at the point of maximum opening. At the onset of the jaw-opening phase, the speed of jaw-opening in the vertical direction was 0. The jaw-closing phase was followed by the occlusal phase. The latter phases were demarcated from the jaw-closing phase by the most lateral position of the jaw-closing path.

We previously reported that a mouthful of solid food is swallowed during the first deglutition during mastication, and that any residual food becomes aggregated by the intra-oral structures into a bolus before being swallowed in the last deglutition (Maeda et al., 2020; Kochi et al., 2021). This suggests that the process of bolus formation before the first deglutition occurs is critical. We first measured the masticatory duration between the onset of the first masticatory cycle and the offset of the masticatory cycle immediately before the first deglutition. Deglutition was identified as an advancing whitish appearance on VE images.

For analysis, masticatory duration, number of masticatory cycles, and masticatory rate in this period were compared among the foods using one-way repeated measures ANOVA followed by Tukey’s HSD test for further analysis. Further, masseter and suprahyoid EMG activities per masticatory cycle was also compared using two-way repeated measures ANOVA (masticatory side vs. non-masticatory side, foods) followed by Tukey’s HSD test for further analysis.

**FIGURE 4:**
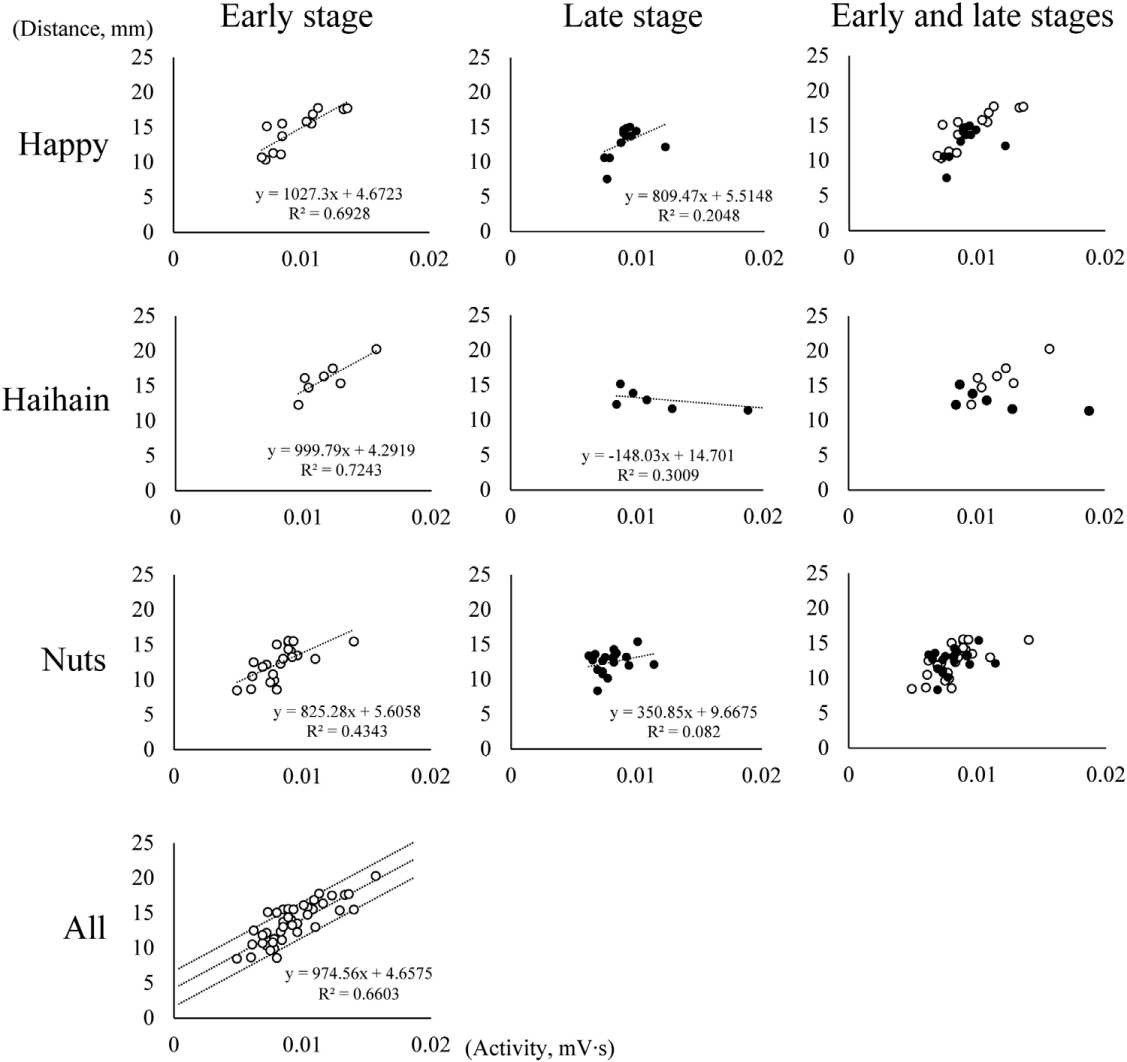
Relationship between suprahyoid electromyographic activity and maximum vertical jaw distance (jaw gape) in one participant. Each graph shows the relationship between suprahyoid activity and jaw gape in one masticatory cycle. The number of masticatory cycles in the masticatory duration was 26 during Happy mastication, 14 during Haihain mastication, and 41 during Nuts mastication with significant positive correlation among them in all test foods in the early stage. Dotted lines indicate the regression line and 95% conference intervals obtained (All).

The relationship between suprahyoid EMG activity and the vertical distance of the mouth (jaw gape) per masticatory cycle was examined using data obtained during mastication before the first deglutition. Because the suprahyoid muscles are known to contribute to jaw-opening during rhythmic mandibular movements, the correlation coefficient (CC) between them in masticatory duration was first calculated in each task for each participant. Unexpectedly, in some participants there was no statistically significant positive correlation in some tasks, which suggested that the suprahyoid muscles did not mainly function for jaw-opening. Subsequently, we divided the masticatory period until the first deglutition into two stages, namely early and late stages depending on the number of masticatory cycles. After collating all the data, including Happy, Haihain, and Nuts mastication in each participant, the CC in the early stage was also calculated for each participant. Because significantly high positive correlation was noted in the early stage in all cases, the regression line and 95% confidence intervals were obtained (Supplementary Table S1, Figure 4). Figure 4 shows the plotted data for one representative participant. Although the plotted data were few during Haihain mastication, there was a clear significant positive correlation among them in the early stage for all test foods; this was also the case for all participants (data not shown). If the plotted data was located between the intervals, we determined that the suprahyoid muscles were activated mainly for jaw-opening. In contrast, if the data was plotted right to the intervals, we determined that the suprahyoid muscles were activated mainly for bolus formation. The former cycle was designated the jaw-opening dominant cycle and the latter the deviation-dominant cycle. The number of these cycles was compared using two-way repeated measures ANOVA on ranks (masticatory side vs. non-masticatory side, among foods) followed by Tukey’s HSD test for further analysis. In addition, the rate of occurrence of these cycles in the masticatory duration was compared in the same manner.

Finally, the magnitude of suprahyoid EMG activity was examined. In each task, suprahyoid EMG activity was divided by jaw gape in each masticatory cycle, and was designated modified suprahyoid activity. For each food, the mean modified suprahyoid activity was compared using two-way repeated measures ANOVA (early vs. late, masticatory vs. non-masticatory side) followed by Tukey’s HSD test for further analysis.

### 2.6.3 Statistical Analysis

The sample size was calculated using G*Power 3.1 30, indicating that at least 18 healthy participants with complete data sets would be needed to achieve a statistical power of 95% and a *p*-value of <0.05, assuming an effect size of 40%. Statistical analysis was performed using SigmaPlot software (SigmaPlot 13.0, Systat Software Inc., San Jose, CA) and BellCurve for Excel (Social Survey Research Information Co., Ltd., Tokyo, Japan). *p* values <0.05 were considered significant. All values are expressed as mean ± SD except those for modified suprahyoid activity (mean ± SEM).

## 3 Results

All participants performed the masticatory task and did not report any discomfort.

### 3.1 Suprahyoid EMG Activity During Tongue Pressure Generation

**FIGURE 5:**
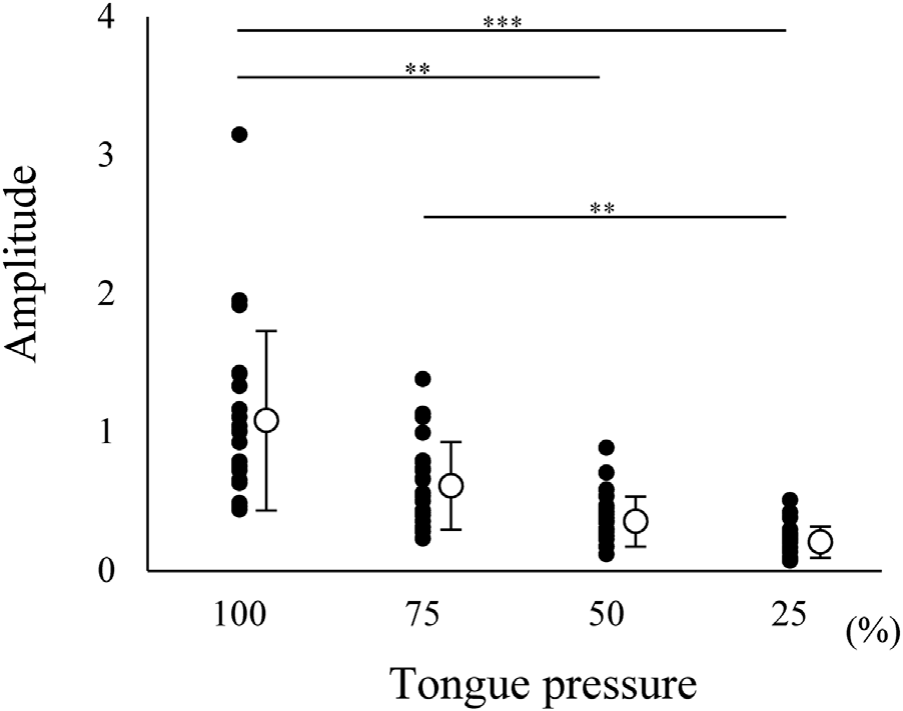
Normalized amplitude of electromyographic burst of suprahyoid muscles during tongue pressure generation at 25, 50, 75, and 100% maximum effort. Open circles indicate mean values. Each value was normalized to that during maximum jaw-opening for each participant. Suprahyoid activity increased with increasing tongue pressure; 0.247 ± 0.114 for 25%, 0.396 ± 0.180 for 50%, 0.650 ± 0.313 for 75%, 1.112 ± 0.644 for 100%. ***p < 0.001, **p < 0.01.

Suprahyoid EMG activity was measured during tongue pressure generation at several force levels, ranging from 25 to 100%. Suprahyoid EMG activity increased with increasing tongue pressure (Figure 5). This indicated that the suprahyoid muscles contribute to elevating the tongue body and/or generating tongue pressure against the hard palate.

### 3.2 General Feature of EMG Activity During Mastication

Representative data of EMGs during unilateral Happy mastication on the habitual side are shown in Figure 3. After mastication started, masticatory rate gradually decreased in the late stage towards the first deglutition. It was apparent that the rhythmic pattern of masseter and suprahyoid EMG burst in the early stage was stable on both sides, and reciprocal masseter and suprahyoid EMG bursts were observed. In the late stage, however, reciprocal patterns were sometimes collapsed such that considerable activity was observed in the suprahyoid EMG activity during the jaw-closing phase (Figure 3B).

**FIGURE 6:**
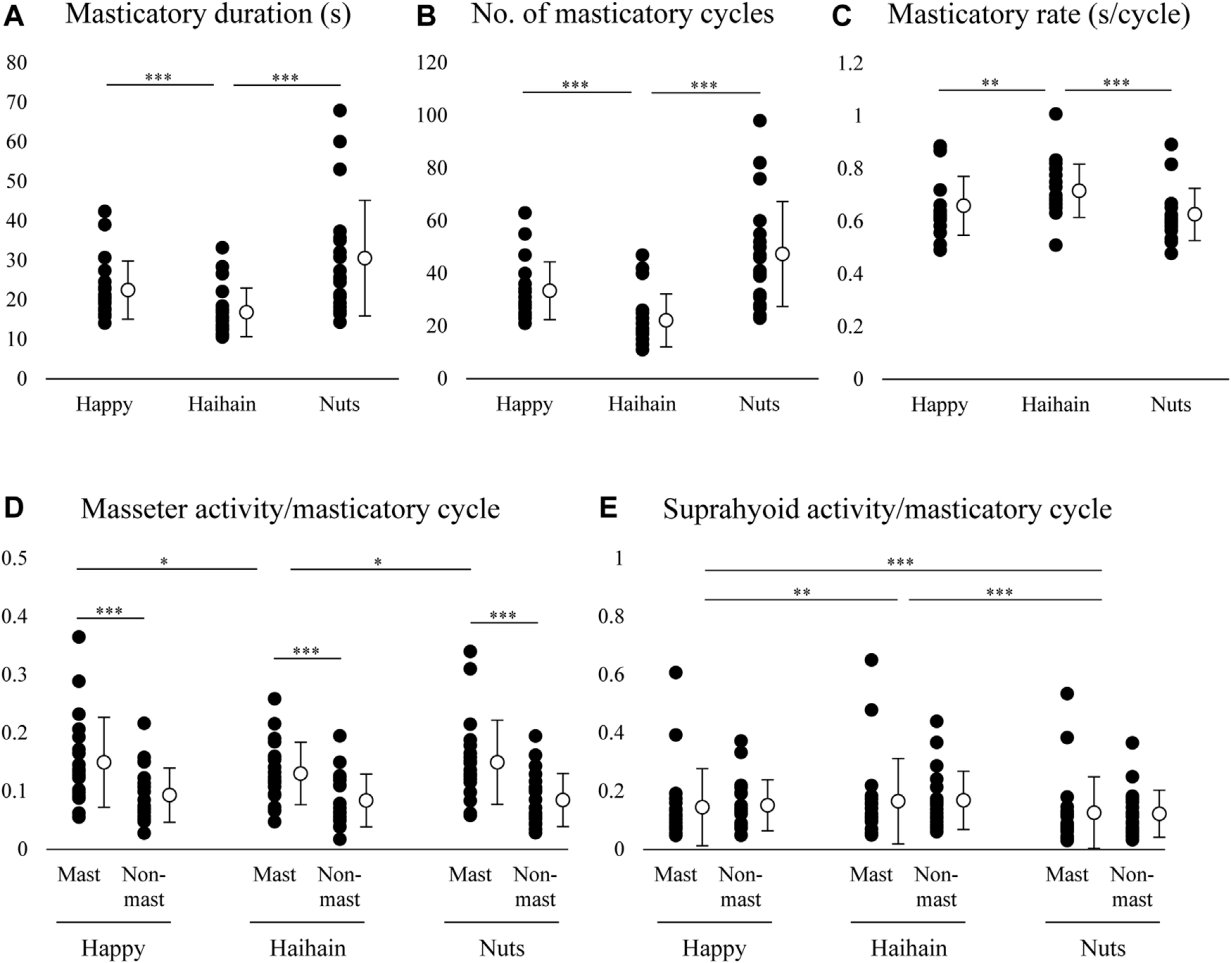
Masticatory duration, number of masticatory cycles, masticatory rate, masseter and suprahyoid electromyographic activity per masticatory cycle. Open circles indicate mean values. **(A)** Masticatory duration was significantly different between Happy and Haihain mastication and between Haihain and Nuts mastication. **(B)** Significant difference in the number of masticatory cycles between Happy and Haihain mastication and between Haihain and Nuts mastication. **(C)** Significant difference in the masticatory rate between Happy and Haihain mastication and between Haihain and Nuts mastication. **(D)** Significantly higher masseter activity on the masticatory side (Mast) than that on the non-masticatory side (Non-mast). There was also a significant difference on the masticatory side between Happy and Haihain and between Haihain and Nuts. **(E)** Difference in suprahyoid activity noted between Happy and Haihain, between Happy and Nuts, and between Haihain and Nuts. There was no difference between the sides. ***p < 0.001, **p < 0.01, *p < 0.05.

For the masticatory duration, number of masticatory cycles, and masticatory rate, one-way repeated measures ANOVA revealed significant difference between Happy and Haihain (*p* < 0.001 for masticatory duration, *p* < 0.001 for number of masticatory cycles, *p* = 0.003 for masticatory rate) and between Haihain and Nuts (*p* < 0.001) (Figures 6A–C).

We also compared the masseter and suprahyoid EMG activity per masticatory cycle among the conditions. For masseter EMG activity, two-way repeated measures ANOVA with Masticatory side × Food revealed a significant main effect of Side (F1, 19 = 25.738, *p* < 0.001) with significant interaction (F2, 38 = 8.362, *p* < 0.001) (Figure 6D). On further post-hoc testing, masseter EMG activity was significantly higher on the masticatory side than that on the non-masticatory side (*p* < 0.001). In addition, masseter EMG activity on the masticatory side was significantly lower during Hahain mastication than Happy and Nuts mastication (*p* = 0.013 for Happy, *p* = 0.014 for Nuts). Regarding suprahyoid EMG activity, a significant main effect was noted only in Food (F2, 38 = 29.575, *p* < 0.001) without significant interaction (F2, 38 = 2.484, *p* = 0.097) (Figure 6E). On post-hoc testing, suprahyoid EMG activity during Nuts mastication was significantly lower than that during Happy and Haihain mastication (*p* < 0.001). Suprahyoid activity during Happy mastication was also significantly lower than that during Haihain mastication (*p* = 0.005).

Taken together, these results indicate that the harder the food, the longer the masticatory duration. The change in duration was generally dependent on changes in the number of masticatory cycles but not on the masticatory rate; the masticatory rate during Haihain mastication was significantly higher than Happy and Nuts mastication. It can also be presumed that the difference in masseter EMG activity was affected by the initial hardness of food or by masticatory behaviors in that the harder the food the higher the masseter EMG activity; this was apparent on the masticatory side. Conversely, suprahyoid EMG activity was significantly higher during Haihain mastication than Happy and Nuts mastication. Contrary to masseter EMG activity, no difference in suprahyoid EMG activity was observed between the sides. These results suggest that suprahyoid EMG activity was neither dependent on the initial hardness of the food nor on mastication side.

### 3.3 Correlation Between Suprahyoid EMG Activity and Jaw Gape

**FIGURE 7:**
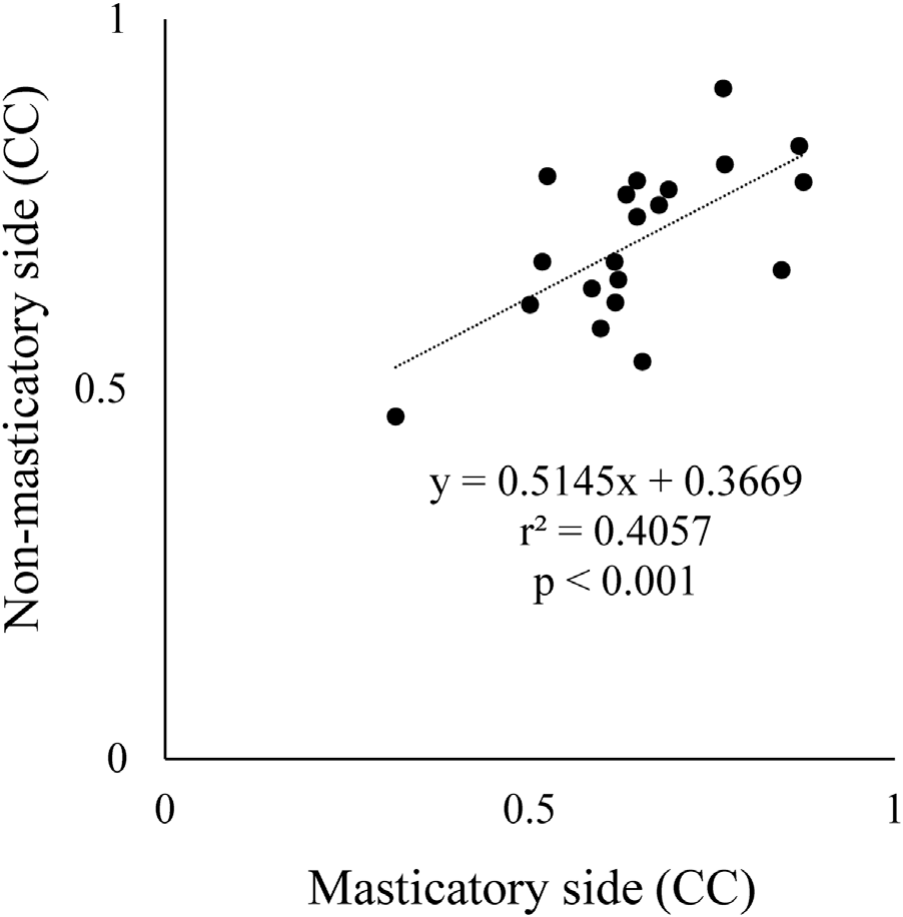
Comparison of correlation coefficient (CC) between masticatory and non-masticatory sides. *X* and *Y* axes indicate CC data between suprahyoid muscle activity and vertical jaw distance per masticatory cycle on masticatory side and non-masticatory sides, respectively. These were significantly correlated with each other.

The CC between suprahyoid EMG activity and jaw gape per masticatory cycle was obtained for each food. As mentioned above, in some participants there was no significant correlation between these parameters on both the masticatory and non-masticatory sides, however, a strongly significant positive correlation was noted in the early stage on both sides. Further, a significant correlation of CC was also observed between the masticatory and non-masticatory sides (Figure 7). These results suggest that the suprahyoid muscles contribute not only to jaw-opening but also to other functions such as bolus formation, and at least in the early stage, these muscles mainly function for jaw-opening on both sides.

**FIGURE 8:**
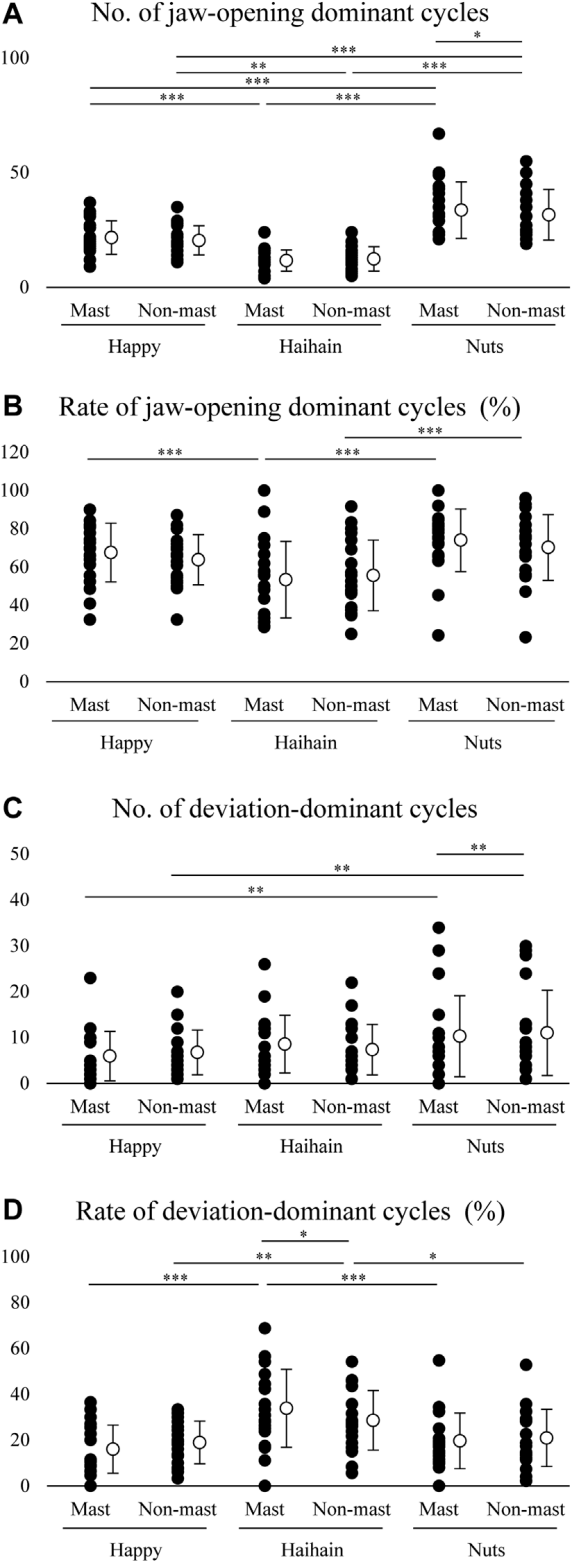
Number and rate of jaw-opening dominant cycles and deviation-dominant cycles. Open circles indicate mean values. **(A)** Significantly higher number of jaw-opening dominant cycles during Nuts mastication than Haihain mastication. This difference was observed between the masticatory (Mast) and non-masticatory sides (Non-mast) during Nuts mastication. Food effect, *p* < 0.001; Food × Side effect, *p* = 0.002. **(B)** Rate of jaw-opening dominant cycles was significantly higher during Nuts and Happy mastication than Haihain mastication on the masticatory side, and during Nuts mastication than Haihain mastication on the non-masticatory side. Food effect, *p* < 0.001; Food × Side effect, *p* = 0.012. **(C)** Significantly higher number of deviation-dominant cycles during Nuts mastication than Happy mastication on both sides. This difference was also observed between the masticatory and non-masticatory sides during Nuts mastication. Food effect, *p* < 0.001; Food × Side effect, *p* = 0.021. **(D)**. Significantly higher rates of deviation-dominant cycles were seen during Haihain mastication than Happy and Nuts mastication on both sides. This difference was also observed between the masticatory and non-masticatory sides during Haihain mastication. Food effect, *p* < 0.001; Food × Side effect (Free vs. Habitual), *p* = 0.005. ***p < 0.001, **p < 0.01, *p < 0.05.

To clarify the function of suprahyoid EMG activity pattern during mastication, we plotted suprahyoid EMG activity and maximum jaw-opening distance (maximum jaw gape) per masticatory cycle. We counted the number of jaw-opening dominant cycles, the data plotted between the intervals, and that of deviation-dominant cycles, the data plotted right to the intervals, in the masticatory duration for each food. For the number of jaw-opening dominant cycles, two-way repeated measures ANOVA with Food × Side (masticatory side vs. non-masticatory side) revealed a significant main effect of Food (F2, 38 = 38.745, *p* < 0.001) but not Side (F1, 19 = 1.307, *p* = 0.267) with significant interaction (F2, 38 = 7.704, *p* = 0.002) (Figure 8A). On post-hoc testing, the effect of Food was apparent on both the masticatory and non-masticatory sides (*p* < 0.001 for all but *p* = 0.004 for Happy vs. Haihain with the non-masticatory side). The difference between the sides was observed only during Nuts mastication (*p* = 0.025). The results were as expected because the order of masticatory duration and number of masticatory cycles was Nuts > Happy > Haihain. Regarding the occurrence rate of jaw-opening dominant cycles, two-way repeated measures ANOVA with Food × Side revealed a significant main effect of Food (F2, 38 = 13.441, *p* < 0.001) but not Side (F1, 19 = 0.704, *p* = 0.412) with significant interaction (F2, 38 = 4.931, *p* = 0.012) (Figure 8B). Post-hoc testing revealed a significantly lower occurrence rate of jaw-opening dominant cycle on the masticatory side in Haihain than Happy (*p* < 0.001) and Nuts (*p* < 0.001). In addition, that on the non-masticatory side was also significantly lower in Haihain than in Nuts (*p* < 0.001).

For the number of deviation-dominant cycles, two-way repeated measures ANOVA with Food × Side revealed a significant main effect of Food (F2, 38 = 7.800, *p* < 0.001) but not Side (F1, 19 = 0.034, *p* = 0.856) with significant interaction (F2, 38 = 4.299, *p* = 0.021) (Figure 8C). On post-hoc testing, a significantly higher number of deviation-dominant cycles was noted both on the masticatory and non-masticatory sides in Nuts than Happy (*p* = 0.002 for both). In addition, that during Nuts mastication was also significantly higher on the non-masticatory side than that on the masticatory side (*p* = 0.008). Regarding the occurrence rate of deviation-dominant cycles, two-way repeated measures ANOVA with Food × Side revealed a significant main effect of Food (F2, 38 = 18.661, *p* < 0.001) but not Side (F1, 19 = 0.044, *p* = 0.836) with significant interaction (F2, 38 = 6.074, *p* = 0.005) (Figure 8D). Post-hoc testing revealed significantly higher occurrence rate of deviation-dominant cycles on both the masticatory and non-masticatory sides in Haihain than in Happy (*p* < 0.001 on the masticatory side, *p* = 0.002 non-masticatory sides) and Nuts (*p* < 0.001 on the masticatory side, *p* = 0.016 non-masticatory sides). In addition, that during Haihain mastication was also significantly higher on the masticatory side than that on the non-masticatory side (*p* = 0.021).

**FIGURE 9:**
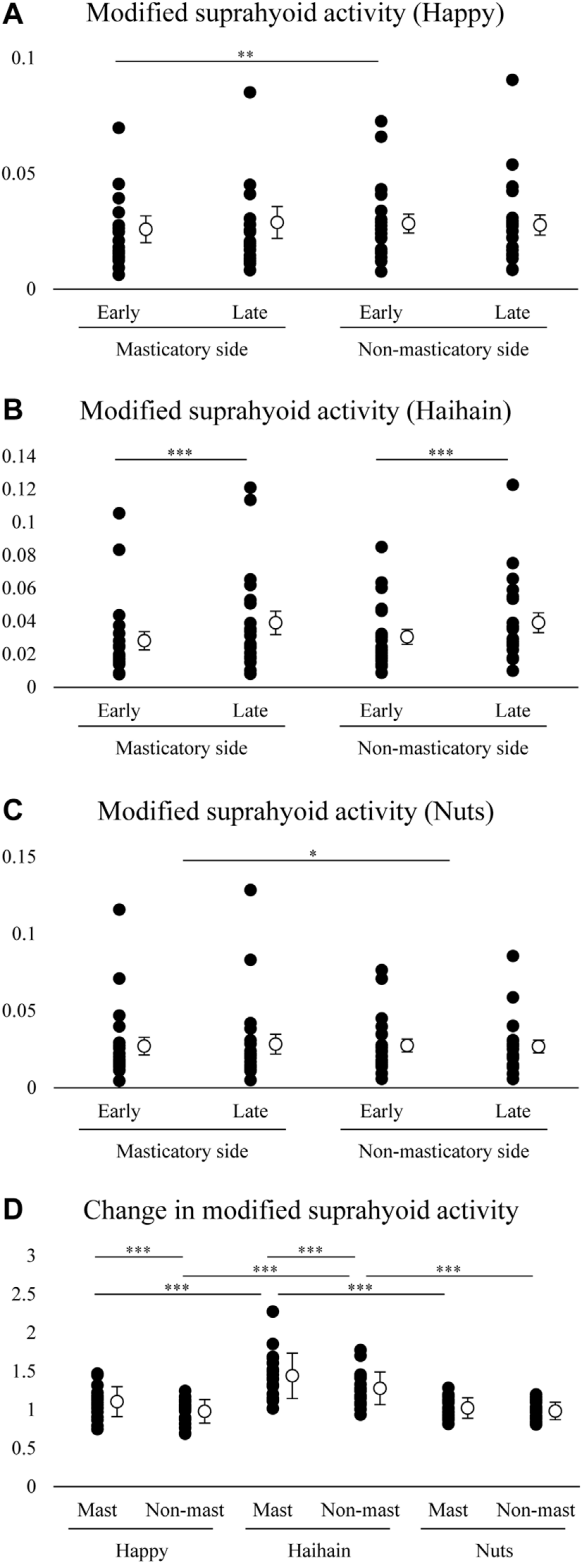
Modified suprahyoid activity and changes in modified suprahyoid activity. Open circles indicate mean values. Modified suprahyoid activity was defined as suprahyoid electromyographic activity divided by maximal vertical jaw distance per masticatory cycle. There was no significant difference between early and late stages on both sides during Happy **(A)** and Nuts **(C)** mastication but during Haihain mastication **(B)**. Side effect, *p* = 0.017; Food × Stage effect, *p* = 0.019 for A. Stage effect, *p* < 0.001 for B. Side effect, *p* = 0.034 for C. **(D)** Increasing rate of modified suprahyoid activity was significantly higher on both the masticatory (Mast) and non-masticatory sides (Non-mast) during Haihain mastication than Happy and Nuts. The difference between the sides was observed during Happy and Haihain mastication. Food effect, *p* < 0.001; Food × Side effect, *p* = 0.005. ***p < 0.001, **p < 0.01, *p < 0.05.

We further compared the modified suprahyoid EMG activity between the sides and between the early and late stages in each food. For Happy, two-way repeated measures ANOVA with Side × Stage (early vs. late) revealed a significant main effect of Side (F1, 19 = 6.808, *p* = 0.017) but not Stage (F1, 19 = 0.816, *p* = 0.378) with significant interaction (F1, 19 = 6.544, *p* = 0.019) (Figure 9A). Post-hoc testing revealed significantly higher suprahyoid EMG activity at the early stage during Happy mastication on the non-masticatory side than on the masticatory side (*p* = 0.005). For Haihain, two-way repeated measures ANOVA revealed a significant main effect of Stage (F1, 19 = 28.267, *p* < 0.001) but not Side (F1, 19 = 3.461, *p* = 0.078) without significant interaction (F1, 19 = 2.835, *p* = 0.109) (Figure 9B). On further post-hoc testing suprahyoid EMG activity at the early stage was significantly lower than that at the late stage on both sides (*p* < 0.001). For Nuts, two-way repeated measures ANOVA revealed a significant main effect of Side (F1, 19 = 5.237, *p* = 0.034) but not Stage (F1, 19 = 0.033, *p* = 0.858) without significant interaction (F1, 19 = 1.495, *p* = 0.236) (Figure 9C). On post-hoc testing, suprahyoid EMG activity on the masticatory side was significantly higher than that on the non-masticatory side (*p* = 0.034).

Finally, changes in modified suprahyoid EMG activity were compared among the foods and between the masticatory and non-masticatory sides. Two-way repeated measures ANOVA with Food × Side revealed a significant main effect of Food (F2, 38 = 22.814, *p* < 0.001) and Side (F1, 19 = 19.303, *p* < 0.001) with significant interaction (F2, 38 = 4.698, *p* = 0.015) (Figure 9D). On post-hoc testing, the increasing rate of suprahyoid EMG activity on both the masticatory and non-masticatory side was significantly higher in Haihain than Happy (*p* < 0.001 both on the masticatory and non-masticatory sides) and Nuts (*p* < 0.001 both on the masticatory and non-masticatory sides). In addition, that on the masticatory side was also significantly higher in Happy and Haihain than that on the non-masticatory side (*p* < 0.001 for both).

## 4 Discussion

### 4.1 Functional Contribution of the Suprahyoid Muscles to Tongue Movements

The process of mastication involves the several intra-oral structures including the lips, tongue, and the hard and soft palates, which function for intake and size-reduction of ingested food *via* the mastication-related muscles. Specifically, the tongue plays a critical role in that it generates pressure against the palate to move the food bolus from side to side within the oral cavity or to propel it posteriorly for deglutition (Logemann, 2014). Further, patterns of tongue movements adjust to sensory information based on location and physical properties such as hardness, cohesiveness, or viscosity of the bolus from cycle to cycle. As with the tongue, the suprahyoid muscles help in manipulating the bolus. When the tongue muscles are activated to form the bolus, the base of the tongue may also need to be elevated and rotated to collect the food together with saliva and/or keep the food positioned in the oral cavity with the cheeks (Abd-El-Malek, 1955). In other words, the suprahyoid muscles must be activated during mastication not only for jaw-opening but also for bolus formation.

Our findings have shown increased suprahyoid EMG amplitude with increasing magnitude of tongue pressure such that maximum tongue pressure generation resulted in larger amplitude suprahyoid EMG bursts, almost the same as that generated during maximum jaw-opening. This strongly suggests that suprahyoid EMG bursts function for both jaw-opening and tongue elevation.

It is possible that the suprahyoid EMG activities were not an accurate outcome measure of contraction of these muscles but also included recordings of activity in surrounding muscles such as the muscles of the tongue, because of the proximity of these muscles to the suprahyoid muscles. In this regard, Palmer et al. (1999) recorded EMG activity from the mylohyoid, anterior belly of the digastric, geniohyoid, and genioglossus muscles and found that the contributions of the genioglossus to EMG activity of the other muscles were quite minimal. Further, we previously demonstrated that the surface EMG activity patterns of the genioglossus and suprahyoid muscles differed from each other in terms of function (Tsukada et al., 2009). Thus, the possibility of contamination of these signals can be excluded.

### 4.2 Difference in Performance Among Test Foods

The muscles of mastication are broadly divided into jaw-closers and jaw-openers. Jaw-closer muscles consist of the masseter, temporalis, and medial pterygoid muscles. These muscles do most of the work of mastication during the jaw-closing power stroke. In contrast, the suprahyoid muscles, which include the mylohyoid, anterior belly of the digastric, and the geniohyoid muscles, are known as the jaw-opener muscles that depress the mandible. Human studies typically utilize surface masseter and suprahyoid EMG activity, due to preferred non-invasiveness of the procedures and ease of recordings. These represent jaw-closer and jaw-opener muscle activity although many other muscles such as the lingual and facial muscles are activated as well (Palmer et al., 1992; Yamada et al., 2005). Therefore, we recorded both masseter and suprahyoid surface EMG activities during mastication in this study.

It is well-known that masticatory behavior adapts to changes in the hardness of the bolus or particle size resulting in altered numbers of masticatory cycles, sequence duration, and masticatory EMG activity (Diaz-Tay et al., 1991; Takada et al., 1994; Hiiemae et al., 1996; Peyron et al., 1997; Miyawaki et al., 2001; Anderson et al., 2002; Peyron et al., 2002; Foster et al., 2006). Thus, the harder or larger the bolus, the more the masticatory cycles, the longer the sequence duration, and the greater the masseter EMG activity, especially on the masticatory side. Our current results were quite consistent with theirs in that masticatory duration, number of masticatory cycles, as with masseter EMG activity were dependent on the initial hardness.

In this study, masticatory rate was significantly higher during Haihain mastication than Happy and Nuts mastication; and suprahyoid activity was significantly higher during Haihain mastication than Happy and Nuts. In our previous study, it was found that the difference in masticatory rate was caused by the difference in suprahyoid activity among the foods (Takei et al., 2020). In addition, we suspect that because of the small size of one piece of Nuts, participants were not required to open the mouth widely during mastication, which led to relatively low masticatory rates. This raises the question as to why the masticatory rate differed between Happy and Haihain. As described above, Happy characteristically had a high fat content as compared with Haihain. We previously found that the water absorption rate and water content were higher for Haihain than Happy, which might affect the force required to move the food bolus in the late stage of mastication (Takei et al., 2020). Thus, the difference in suprahyoid activity patterns among the foods may not have resulted from only one property.

Further, there was no difference in suprahyoid activity between the masticatory and non-masticatory sides. The suprahyoid muscle group includes the mylohyoid, anterior belly of the digastric, and the geniohyoid muscles; the geniohyoid functions in jaw-opening. In addition, they also function with the tongue muscles to form the bolus during mastication (Khan and Bordoni, 2021). Considering the results obtained from EMG data in that suprahyoid activity differed among the foods but not between sides or between masticatory tasks, suprahyoid muscle activity does not seem to be affected by masticatory side. However, these results were obtained from EMG data only. We therefore simultaneously recorded mandibular kinematics in our experiment (see next section).

### 4.3 Difference in Sequence Changes Among Foods

The suprahyoid muscles, particularly the anterior belly of the digastric and the mylohyoid, dominantly contribute to jaw-opening (Khan and Bordoni, 2021). The digastric muscle helps in depressing and retracting the mandible functionally but is less involved in deglutition, at least in animals (Doty and Bosma, 1956; Tsujimura et al., 2012). Conversely, the mylohyoid muscle also functions to elevate the floor of the mouth and the tongue during deglutition or speaking while the geniohyoid muscle contributes to upward and forward movements of the hyoid, and hence widening of the passage for the bolus during deglutition. Thus, assessment using only EMG data makes it difficult to precisely identify the functional role of each EMG burst during mastication.

A major focus of our study was to clarify the possibility of determining how the suprahyoid muscles function throughout the masticatory sequence. During mastication, the food bolus is manipulated differently depending on the masticatory stage, early, middle, or late. In the early stage, jaw-closing muscles mainly participated in reducing bolus size, and the food bolus hardness rapidly decreased (Iguchi et al., 2015; Maeda et al., 2020; Kochi et al., 2021). Conversely, in the late stage the tongue and suprahyoid muscles are dominantly activated possibly to gradually alter bolus properties such as adhesiveness or cohesiveness by mixing it with saliva (Peyron et al., 2011; Maeda et al., 2020; Takei et al., 2020; Kochi et al., 2021). From this perspective, we hypothesized that the role of the suprahyoid muscles differed between the early and late stages. In fact, there was an excellent positive correlation between suprahyoid activity and jaw gape in the early stage during all mastication in all participants. We therefore decided to use the suprahyoid activity/jaw gape per masticatory cycle ratio in the early stage as a reference. When the suprahyoid muscles mainly contribute to elevating the floor of the mouth and the tongue for bolus formation, the ratio must increase. Thus, we compared the number and occurrence ratio of jaw-opening dominant and deviation-dominant phases and the amplitude of the suprahyoid activity/jaw gape ratio among the foods. We found that the suprahyoid muscles primarily function for jaw-opening during Nuts mastication and for bolus formation during Haihain mastication, especially in the late stage.

Numerous studies have used Nuts as a test food to investigate masticatory movements because of its toughness (Wilding and Lewin, 1994; Agrawal et al., 2000; Mishellany et al., 2006; van der Bilt and Abbink, 2017; Capuano et al., 2020). The advantage of using Nuts is likely because peanuts are a naturally hard food with dimensional stability. During mastication, the bolus gradually changes in size and rheological properties such as hardness, cohesiveness, and adhesiveness. Previous studies suggested that deglutition cannot be initiated when bolus particles remain above a certain size, which is considered the deglutition threshold (Feldman et al., 1980; Hutchings and Lillford, 1988; Prinz and Lucas, 1995). Further, the size distribution of the bolus particles is a determining factor in making the bolus sufficiently cohesive to enable deglutition to occur safely (Mishellany et al., 2006). Thus, the focus of masticatory performance using Nuts might be the breakdown of the bolus by the masticatory muscles. It can be concluded that the suprahyoid muscles were dominantly activated for jaw-opening during Nuts mastication regardless of the side although a minor but significant difference was noted between sides.

As compared with Happy mastication in which the deviation-dominant phase was relatively shorter, during Haihain mastication, the number of deviation-dominant phases was not significantly lower and the rate of deviation-dominant phases was significantly higher than Happy and Nuts even though the number of masticatory cycles was lowest during Haihain mastication. Further, during Haihain mastication, modified suprahyoid activity was significantly higher in the late than in the early stage on both the masticatory and non-masticatory sides; a difference was noted between the masticatory and non-masticatory sides. In a previous study, we found that the increasing ratio of masticatory cycle time in the late stage was highest in Haihain mastication vs. the other rice cracker suggesting that the longer masticatory cycle time and higher suprahyoid activity can be attributed to the water absorption rate of the bolus, and not hardness (Takei et al., 2020). That study also found that there was no difference in the adhesiveness and cohesiveness of the bolus at deglutition initiation between Happy and Haihain. These results suggest that bolus properties, as well as oral conditions such as dryness, significantly affect suprahyoid activity. A negative impact of oral dryness on mastication can thus be presumed. Shinkawa et al. (2009) reported that poor masticatory ability is associated with lower mucosal moisture in elderly individuals. These results suggest the need to consider that masticatory behavior is affected by both bolus properties and oral conditions such as salivary flow rate or oral dryness.

To our knowledge, ours is the first report to have demonstrated the difference in the contribution of the suprahyoid muscles to bolus formation between the masticatory and non-masticatory sides. Previous studies introduced a new method to record neck surface EMG activities, which represents the force of posterior tongue lifting (Manda et al., 2016; Mori et al., 2021). The authors demonstrated that neck EMG activity was significantly higher on the masticatory side than on the non-masticatory side although there was no difference among the stages. Our findings in this study are partly consistent with theirs in that the EMGs recorded on the masticatory side dominantly contributed to bolus manipulation during mastication. In their study, however, they evaluated only the peak amplitude of EMG bursts. In addition, as described, the suprahyoid muscles are activated not only during jaw opening but also during tongue-lifting. Because particle size gradually decreases in a masticatory sequence, it is vital to consider how the muscle activity changes with changes in bolus size and jaw gape during mastication. In this respect, it is noteworthy that the change in modified suprahyoid activity was observed only during Happy and Haihain mastication in this study. Future studies, should precisely clarify which conditions determine the asymmetry of these functions.

### 4.4 Limitations

Several limitations of this study should be considered when interpreting the findings. First, we recruited only healthy young male and female participants, and so these findings could not be generalized to other/older populations. Considering the effects of age, particularly regarding the effects of dental status or oral dryness, recruiting other populations would help clarify how these conditions affect masticatory behaviors. In addition, gender difference should also be considered. Although our previous study reported difference in the masticatory function between the genders (Maeda et al., 2020), we believe that the nature of masticatory movements is not much different between them. In our future study, we will focus on the effect of age and gender on the masticatory kinematics as well as EMG activity. Second, only two rice crackers and peanuts were used, and so we could not determine specifically which factors, including the shape, size, or taste of the foods, were critical for determining the masticatory movements. Third, although the focus was on only jaw gape to determine the function of the suprahyoid muscles, other parameters such as the duration or speed of mandibular movements should also be evaluated. Although there was a mild correlation between EMG activity and the duration of each phase, the CC was always lower than that between EMG activity and jaw gape (data not shown). Fourth, we analyzed the EMG signals using only the area under the curve, but did not consider timing such as onset, offset, and peak time nor the changes in these values between conditions. Fifth, we did not directly visualize bolus transport in the oral cavity and pharynx. Our future study will involve simultaneous recordings of EMG activity and imaging.

Despite these limitations, our findings clearly demonstrate a functional role of the suprahyoid muscles during mastication of solid foods with different initial consistencies by analyzing both EMG activity and mandibular kinematics. This provides a useful modality for evaluating the masticatory physiology of a range of solid foods.

## 5 Conclusion

This study showed the difference in suprahyoid EMG activity during mastication of solid foods with different initial properties. We demonstrated that the suprahyoid muscle activity increased not for jaw opening, but for bolus formation especially on the masticatory side during the late stage of soft rice cracker (Haihain) mastication. These findings were obtained from assessments using both EMG activity and mandibular kinematic recordings. In a clinical situation, not only hardness but also other characteristics of the solid food should be considered to evaluate masticatory function.

## Data Availability

The original contributions presented in the study are included in the article/Supplementary Material, further inquiries can be directed to the corresponding author.
