# Supplementary material for: Functional Role of Suprahyoid Muscles in Bolus Formation During Mastication
Source: Front Physiol. 2022 Jun 8;13:881891. doi: 10.3389/fphys.2022.881891 (PMC9214202; doi:10.3389/fphys.2022.881891)
Supplement: Supplementary file 1 [file Table1.docx]

**Supplementary table　Correlation between suprahyoid activity and maximum vertical jaw distance per masticatory cycle in the early stage CC**

Note: CC, Correlation coefficient; Mast, masticatory side; Non-mast, non-masticatory side; Sub, subject.
